# Fusion of multi-scale geometric features and frequency domain decomposition for stereo matching network

**DOI:** 10.1371/journal.pone.0340473

**Published:** 2026-01-16

**Authors:** Hua Hou, Diancheng Wang, Jinqian Xu, Yan Wang

**Affiliations:** School of Information and Electrical Engineering, Hebei University of Engineering, Handan, China; King Abdulaziz University Faculty of Engineering, SAUDI ARABIA

## Abstract

In learning-based stereo matching methods, a feature information-rich and concise cost volume is crucial for achieving high-precision and high-efficiency stereo matching. Aiming at the problem that the cost volume lacks global geometric information, which leads to confusing foreground and background disparity estimation and blurring at edges and details, this paper proposes a fusion of multi-scale geometric features and frequency domain decomposition stereo matching network. Firstly, the initial cost volume is processed by the multi-scale geometric extraction module, which achieves an effective conversion from local correlation to global geometric information understanding, and significantly enhances the perception of scene boundaries and occluded regions. In the cost aggregation stage, we introduce an adaptive guidance mechanism based on channel attention, which not only improves the cost aggregation efficiency but also reduces the time overhead. In the disparity refinement stage, we not only use the iterative update of disparity based on multi-scale GRU, but also introduce the high and low-frequency separation of disparity reconstruction network, which reconstructs the disparity by decomposing the high and low-frequency errors, and is able to obtain a finer full-resolution disparity map. Our method achieves state-of-the-art performance on benchmark tests across multiple datasets, including Scene mFlow, KITTI2012, KITTI2015, ETH3D, and Middlebury. Compared to mainstream approaches, our method demonstrates excellent results on the KITTI2015 test set, attaining error rates of 1.39% in the background region (D1-bg) and 2.54% in the foreground region (D1-fg), while maintaining real-time inference capabilities.

## 1 Introduction

Stereo matching has been of great interest in the field of computer vision because of its ability to infer 3D scene geometric information from captured images [1], and is widely used in 3D reconstruction [2], medical imaging [3], augmented reality [4], and autonomous driving [5]. Given a pair of rectified stereo images, the goal of stereo matching is to compute the disparity for each pixel in the reference image, thereby generating a disparity map. Disparity refers to the horizontal displacement between a pair of corresponding pixels in the left and right images [6]. For a point with pixel coordinate (*h*,*w*) in the left image, if its corresponding point can be found in the right image with coordinate (*h*–*d*,*w*), then the depth of this pixel point can be calculated by the formula $\frac{B\cdot f}{d}$, where *f* is the focal length of the camera and *B* is the length of the baseline.

Traditional stereo matching methods are divided into four steps:matching cost computation, cost aggregation, disparity estimation, and disparity refinement [7], which lays the foundation for deep estimation. In recent years, deep learning has developed rapidly, and stereo matching methods based on deep learning have shown significant advantages with higher accuracy, better robustness and stronger generalisation. Zbontar and LeCun firstly applied Convolutional Neural Network (CNN) to the depth estimation task, and proposed a non-end-to-end stereo matching network MC-CNN [8]. This network replaces the cost calculation step in the traditional method with a deep learning-based cost calculation method, and improves the accuracy of disparity estimation by learning more effective feature information, which breaks through the limitations of manual calculation in the traditional method, but the non-end-to-end method lacks the complexity of global information and is computationally complicated, which has certain limitations. To solve this problem, researchers have proposed end-to-end stereo matching networks. DispNet [9] is the earliest end-to-end stereo matching network, the whole network can automatically learn feature representations and directly generate fine disparity maps. DispNet performs the cost aggregation by 2D convolution. AANet [10], HITNet [11], Bi3D [12] and SMD-Nets [13] are all classical 2D convolution-based stereo matching networks. The 2D convolution-based stereo matching network has the characteristics of simple network structure and high operational efficiency, but the 3D cost volume provides limited feature information, and the accuracy and robustness of the disparity prediction need to be further improved.

Stereo matching networks based on 3D convolution show great advantages in high accuracy and high robustness scenarios because 3D convolution is able to aggregate feature information in both spatial and parallax dimensions simultaneously, and the generated 4D cost volume contains more detail information and contextual information. GCNet [14] used 3D convolution for the first time to perform cost-space filtering to achieve the matching cost aggregation, and used soft argmax to compute the disparity, and the 3D convolutional cost filtering and soft argmax proposed in this work have been widely used by subsequent works. GwcNet [15] is a typical 3D convolutional-based stereo matching network, which constructs the cost volume by using a grouped relevance approach, computes the relevance vector after grouping the left and right features, constructs the cost volume, and uses an improved 3D stacked hourglass network for disparity regression. However, 3D convolution requires high computational cost, and how to design a more efficient stereo matching network becomes an urgent problem. Recently, methods based on multiple iterations of optimization have demonstrated excellent performance on both standard benchmarks and high-resolution images. Compared to methods based on 3D convolution for cost aggregation, the iterative approach extracts feature information repeatedly from a high-resolution 4D cost volume, progressively updating the disparity map, thereby circumventing complex cost aggregation operations. Consequently, this method can be directly applied to high-resolution images to generate disparity maps. For instance, RAFT-Stereo [16] utilizes multi-level convolutional Gated Recurrent Units (GRUs) [17] to iteratively update the disparity map by extracting local correlation cues from the correlation volume of all pixel pairs. DLNR [18] introduces a decoupled Long Short-Term Memory (LSTM) [19] that retains more high-frequency information during iterations, producing disparity maps with greater high-frequency details. However, both RAFT-Stereo [16] and DLNR [18] employ a fixed receptive field, making it difficult to simultaneously capture high-frequency information in edge regions and low-frequency information in smooth areas. To address this, Selective-Stereo [20] proposes a Selective Recurrent Unit (SRU), which enables stereo matching networks to aggregate hidden disparity information across multiple frequencies, thereby reducing the risk of losing important hidden disparity information during the iterative process. However, these iterative methods all search for feature information from correlation pixel cost volumes during iterative updates, and these cost volumes rely on local correlations, lacking global geometric and contextual information.

**Fig 1:**
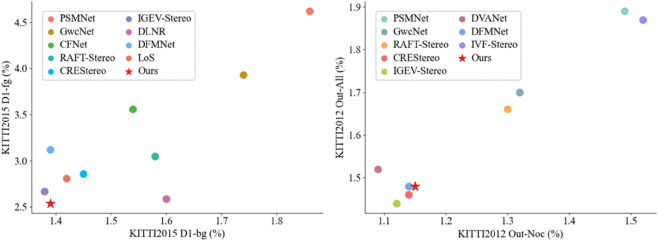
Comparison of MGFD-Stereo with mainstream stereo algorithms at KITTI2015 and KITTI2012. D1-bg and D1-fg are the percentage of three-pixel error for the background and foreground regions, respectively, and the value is as small as possible. Out-Noc and Out-All are the percentage of three-pixel error for the non-obscured pixel region and all pixel region, respectively. The value is also the smaller the better.

In order to solve the above problems, we propose a multi-scale geometric extraction module, which can better aggregate the geometric information in the cost volume in order to enhance the representation of the cost volume, and we introduce a feature-channel-attention-guided cost aggregation method in order to effectively reduce the time overhead during cost aggregation. In order to obtain refined full-resolution disparity maps, we not only use an iterative-based optimisation method, but also combine the idea of frequency domain decomposition to generate accurate disparity maps by separating high-frequency and low-frequency errors for targeted correction. Our proposed Fusion of Multi-Scale Geometric Features and Frequency-Domain Decomposition for Stereo Matching Network (MGFD-Stereo) achieved excellent performance on benchmark tests on the Scene Flow [9], KITTI 2012 [21], and KITTI 2015 [22] datasets. Fig 1 shows the results of the comparison between MGFD-Stereo and mainstream methods on KITTI.

## 2 Related work

### 2.1 Cost volume construction and aggregation

Constructing cost volume is a core aspect of the stereo matching task, which directly affects the accuracy and computational efficiency of the disparity estimation. Recently, significant progress has been made in constructing the cost volume using approaches based on learning. Comprehensive image feature extraction is a key step in building an effective cost volume, and the effectiveness of multi-scale mechanisms in capturing contextual information has been widely validated across multiple domains. For example, ESTINet [23] incorporates a multi-scale strategy in its Spatial Information Collection Module (SICM), where features at different spatial resolutions are extracted and fused to improve the overall quality of video deraining. In the single-image desnowing domain, multi-scale mechanisms have likewise seen broad adoption. DDMSNet [24] introduces multi-scale designs in both the pixel and feature domains, enabling the simultaneous capture of fine-grained textures and global context, thereby further enhancing desnowing restoration performance. GridFormer [25] proposes a grid-structured multi-scale interaction module for image restoration under adverse weather. this module balances global dependencies and local details while effectively controlling computational cost, achieving superior performance and exhibiting high scalability. In addition, MB-TaylorFormer V2 [26] introduces a multi-scale patch embedding module that employs multi-branch architectures, deformable convolutions, and convolutions with different receptive fields to extract feature information, thereby providing information-rich and flexible tokens for subsequent processing.

In the field of stereo matching, PSMNet [6] constructs cost volume at different scales by introducing spatial pyramid pooling and uses stacked hourglass 3D convolutional neural networks to supervise the specification of the cost volume, which is a milestone work. Subsequent CFNet [27] builds upon PSMNet [6] and introduces a cascade cost volume fusion-based network. This network fuses multiple low-resolution cost volumes to generate a feature-rich cost volume, thereby enhancing the robustness of the stereo matching network. However, the cubic computational complexity and high memory consumption of 3D CNNs are very expensive to deploy in practical applications, for this reason, AANet [10] proposed a cross-scale cost aggregation method via sparse point sets and a neural network-based cross-scale cost aggregation algorithm, which efficiently solves the edge diffusion problem and handles texture-free regions. IGEV-Stereo [28] constructs a combined geometric codomain that encodes geometric features and contextual information, and this network can be used to deal with locally blurred regions in images by effectively capturing non-local geometric information. Mocha-Stereo [29] proposed a Motif channel correlation volume for solving the problem of mismatched edge details due to the loss of geometric structural information in the feature channel generation process. DFMNet [30] introduces a cost-filtering volume that leverages guidance weights derived from group-wise correlations to filter redundant information in the concatenated volume, thereby improving the accuracy of feature representations. LoS [31] proposes a novel local structure representation, termed Local Structure Information (LSI), to replace the 3D cost volume, enabling more accurate stereo matching without the need to construct a 3D cost volume. IVF-Stereo [32] addresses the issue of cost volume distortion in asymmetric stereo matching by iteratively fusing complementary correlation volumes and concatenated volumes, combined with multi-peak search and two-stage optimization.

Some researchers introduced the attention mechanism into the stereo matching task, STTR [33] revisited the stereo depth estimation problem from the perspective of sequence-to-sequence correspondence by adding Transformer [34] to a stereo matching network for dense pixel matching alternative cost volume building using positional information and the attention mechanism. ACVNet [35] designed an innovative approach for cost volume construction that uses correlation clues to generate attention weights, suppressing redundant information and enhancing matching-related information. Additionally, it employs multi-layer adaptive patch matching to improve the distinctiveness of the matching cost at different disparity levels.

There are many works devoted to study how to efficiently cost aggregation, CSPN [36] and GANet [37] use spatial transformations to aggregate costs. CSPN [36] learns the affinity of neighbouring pixels through a deep convolutional network to form an affinity matrix, which performs the cost aggregation. GANet [37] proposed semi-global and local guided aggregation layers to replace computationally expensive 3D convolutional layers, thereby achieving efficient and accurate cost aggregation. These methods achieve high accuracy depth estimation at the expense of more time. StereoNet [38], ADCPNet [39] and IINet [40] focus on building real-time stereo matching networks, but these networks often achieve fast depth estimation by sacrificing some accuracy. We use 3D convolution-based spatial filtering, but focuses on both speed and efficiency.

### 2.2 Disparity refinement

Disparity refinement is a key post-processing aspect in stereo matching algorithms, aiming to improve the accuracy and robustness of the initial disparity estimation. In recent years, methods based on iterative optimisation have achieved excellent performance in stereo matching work. Zachary Teed firstly proposed the iterative model RAFT [41], which constructs a 4D cost volume by calculating the relationship between all pixel pairs, and then retrieves iterative features from the correlation volume for optical flow estimation using a GRU-based update operator. Later, Lahav Lipson migrated the RAFT framework to stereo matching by constructing the RAFT-Stereo [16] stereo matching network, which introduces multiple levels of GRU convolution to iteratively update the disparity values by using local surrogate values retrieved from the correlations of all the pixel pairs to improve the accuracy of the disparity estimation. CREStereo [42] improved on RAFT-Stereo by introducing an adaptive group correlation module to improve the generalisation of the network, as well as a recursive updating module and a cascade stacking module, which are used to obtain finer disparity maps. Many stereo matching works proposed specific modules to deal with the initial disparity in order to improve the accuracy of the disparity maps. StereoDRNet [43] proposed a novel disparity refinement network that uses geometric error as input for optimization, effectively addressing the issue of inconsistency in geometric information in the disparity map. Eai-Stereo [44], in response to the blurriness and lack of detail in the generated disparity maps, proposed an error-aware module, which combines the high-frequency information of the original image to improve the error correction capability of the network, thus generating excellent disparity maps. DLNR [18] proposed a disparity normalization refinement module, which, by normalizing the disparity with respect to the image width, addresses the issue of module failure in cross-domain scenarios.

In recent years, frequency domain decomposition has been widely applied in the field of computer vision. HLNet [45] designs a high-low frequency decomposition-based approach for image restoration and enhancement. By introducing high-low frequency decomposition blocks, it can effectively handle different types of image degradation, thereby achieving higher-quality image restoration. HiLoF-GAN [46] is a novel underwater image enhancement method that leverages Generative Adversarial Network (GAN) combined with high-low frequency separation, effectively improving the quality and visual effects of underwater images. In the field of video anomaly detection, FE-VAD [47] incorporates high-low frequency enhancement modules to separate and modulate high-low frequency information in both spatial and temporal domains. We will use a two-stage disparity refinement in order to further improve the accuracy of the disparity map, with the first stage being a multi-scale GRU-based disparity refinement and the second stage being a frequency domain decomposition-based disparity reconstruction.

## 3 Methods

**Fig 2:**
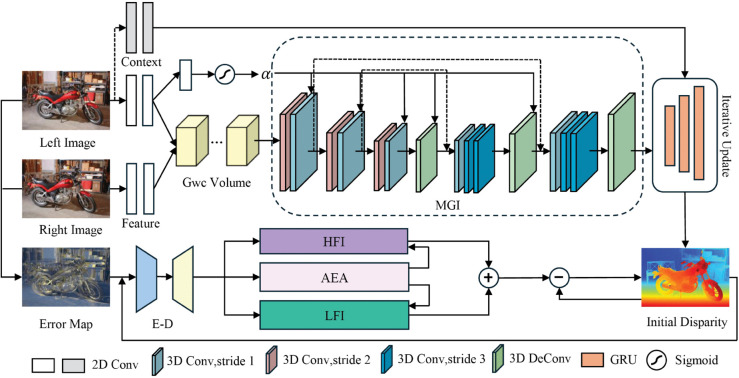
Overview of our proposed MGFD-Stereo. After multi-scale feature extraction, the initial cost volume is constructed using group correlation, and the cost volume is processed by Multi-scale Geometric Information (MGI), which makes the cost volume have more geometric information. In the cost aggregation, we introduce the Feature Channel Attention Guided (FCAG) mechanism to improve the aggregation efficiency and accuracy. Finally, disparity refinement is achieved by iteratively updating the parallax through multi-scale GRU and High and Low Frequency Separation Disparity Reconstruction (HLFS).

In this section, we describe the structure of MGFD-Stereo in detail. The overall framework of our model is shown in Fig 2 and consists of feature extraction, fusion of geometrical information with cost volume and two-stage disparity refinement.

### 3.1 Feature extraction

Feature extraction consists of two parts: 1) a multi-scale feature extraction network that extracts multi-scale features for constructing cost volumes and guiding cost volume aggregation, and 2) a multi-scale context extraction network that extracts multi-scale context features for iterative updating of disparity based on multi-scale GRUs.

Multi-scale Feature Extraction Network:for the corrected left and right images, we first use the MobileNetV2 [48] model pre-trained on ImageNet [49] to extract features, producing feature maps at 1/32 of the original image resolution. We then use an upsampling module with skip connections to progressively restore the feature maps to resolutions of 1/16, 1/8, and 1/4 of the original image, thereby obtaining multi-scale feature maps. We use the 1/4 resolution feature map to construct the cost volume, and 1/4, 1/8, 1/16, and 1/32 resolution images are used to guide the cost aggregation.

Multi-scale Context Extraction Network: this network is applied to the left image only and consists of a series of downsampling layers and residual blocks that generate feature maps at 1/4, 1/8, 1/16 of the input image resolution, each with 128 channels. Contextual features are used to initialise the hidden state of the multi-scale GRU, and during the iterative refinement of disparity, the GRU generates updated hidden states.

### 3.2 Cost volume integrating geometric information

#### 3.2.1 Cost volume construction.

We construct the initial cost volume using the 1/4 resolution feature map of the original image, extracted by the multi-scale feature extraction network. We construct a grouped correlation volume using the GwcNet method, which divides the features $f_{l,4}(f_{r,4})$ into $N_{g}(N_{g}=8)$ groups along the channel dimensions and calculates the correlation volume group by group.

$$V_{gwc}(d,h,w,g)=\frac{1}{N_{c}/N_{g}}\left\langle f_{l,4}^{\;1.5ptg}(h,w),f_{r,4}^{\;1.5ptg}(h-d,w)\right\rangle$$ (1)

Where *N*_*c*_ is the number of feature channels, *d* is the index of disparity, and is the inner product.

$V_{gwc}$ is limited by local similarity due to its dependence on feature correlation and cannot adequately express the global geometric information of an image. Inspired by the fact that 3D convolution can effectively encode geometric information, we propose a Multi-scale Geometric Information (MGI) module that achieves an effective transformation from local correlation to global geometric understanding. The module is an encoder-decoder structure, where the encoder and decoder consist of three downsampling blocks and three upsampling blocks, respectively, with each downsampling block consists of two 3×3×3 convolutions, and each upsampling block consists of a 3D transpose convolution with a kernel size of 4×4×4. $V_{gwc}$ is downsampled to 1/2, 1/4, and 1/8 of the original resolution by the encoder, with the cost volume having 16, 32, and 48 channels at each respective downsampling stage. In the decoder stage, the cost volume is gradually upsampled by the transpose convolution to restore to the original resolution. In order to achieve the effective fusion of multi-scale features, we also introduce a skip connection mechanism to merge the output results of the encoder and into the decoder stage, which can enable the network to simultaneously utilise both shallow and deep features, and to capture both detailed features and deep semantic information, in order to enhance the expressive power of the geometric features of the geometric cost volume.

#### 3.2.2 Feature channel attention-guided cost aggregation.

The cost aggregation method using spatial transformation can effectively capture spatial neighbourhood information, but this method uses a large number of 3D convolutions, takes a long time to compute, and leads to increased memory consumption. For this reason, we introduce a lightweight Feature Channel Attention Guided (FCAG) mechanism, which uses the attention weights generated by the feature maps to dynamically adjust the importance of each channel in the cost volume. For a cost volume $V_{i}\left(i=4,8,16,32\right)$ of size $\frac{H}{i}\times \frac{W}{i}\times \frac{D}{i}$, feature maps of the same resolution are fed into the network, and channel attention weights are generated for each pixel of each channel, which are then applied to the cost volume to guide cost aggregation. We integrate FCAG into the cost aggregation to effectively improve the efficiency of aggregation stage.

$$\alpha =\sigma \left(F^{2D}(f_{l,i})\right)$$ (2)

$$V_{i}^{{\;}^{\prime }}=\alpha ⊙V_{i}$$ (3)

where *σ* is a sigmoid function and ⊙ is realised by 2D convolution.

### 3.3 Disparity refinement

In the stage of disparity refinement, MGFD-Stereo is divided into two stages: the first stage is the iterative refinement of the disparity based on multi-scale GRU, and the second stage is the high and low-frequency error-separated disparity reconstruction.

#### 3.3.1 GRU-based iterative refinement of disparity.

To obtain the initial disparity map, we use the soft max method to perform regression calculations from the cost volume $V_{i}^{{\;}^{\prime }}$, which can be expressed as:

$$d_{0}=\sum_{d=0}^{D-1}d\times Softmax\left(V_{i}^{\prime }(d)\right)$$ (4)

where *d* is a set of discrete disparity indices.

In the iterative update phase of the multiscale GRU-based disparity, we predict a series of disparity fields $\left\{d_{1},d_{2},...,d_{i}\right\}$ starting from the initial disparity *d*_0_ = 0. In each iterative update, a set of relevant features $V_{g}$ are indexed from a correlation pyramid based on the current value of the disparity *d*_*i*_. The correlation pyramid here is a composite geometric pyramid constructed by combining relevant geometric features and relevant pixel features. The correlation features and disparity values are then processed through a encoding layer and concatenated with contextual features to obtain *x*_*i*_, which is input to the multi-scale GRU.

$$x_{i}=[Encoder_{g}(V_{g}),Encoder_{d}(d_{i}),d_{i}]$$ (5)

$$z_{i}=\sigma \left(2DCon\nu \left(\left[h_{i-1},x_{i}\right],W_{z}\right)+c_{i}\right)$$ (6)

$$r_{i}=\sigma \left(2DCon\nu \left(\left[h_{i-1},x_{i}\right],W_{r}\right)+c_{r}\right)$$ (7)

$${\tilde{h}}_{i}=tanh\left(2DCon\nu \left(\left[r_{i}⊙h_{i-1},x_{i}\right],W_{h}\right)+c_{h}\right)$$ (8)

$$h_{i}=\left(1-z_{i}\right)⊙h_{i-1}+z_{i}⊙{\tilde{h}}_{i}$$ (9)

Where *x*_*i*_ is the current input disparity value, *z*_*i*_ is the update gate, *r*_*i*_ resets the gate, and *c*_*i*_, *c*_*r*_, *c*_*h*_ and generate context features for the context feature network.

The hidden state *h*_*i*_ is obtained, which is decoded by the convolutional layer to obtain the disparity, which is used to update the disparity $\Delta d_{i}$ and can be expressed as:

$$d_{i+1}=d_{i}+\Delta d_{i}$$ (10)

#### 3.3.2 High and low frequency separation for disparity refinement.

The iteratively updated disparity map has 1/4 resolution of the original image, which is up-sampled to get the full-resolution disparity map. In order to get a finer disparity map, we propose the High and Low Frequency Separation Disparity Reconstruction (HLFS). We use the error E as the input to this module. The error E is obtained by warping the right image to the left view based on the disparity and then computing the difference with the left image, which can be expressed as:

$$E=warp(I_{r},disp)-I_{l}$$ (11)

Where *disp* is the upsampled disparity map after iterative update, and *warp* is the warping transformation function.

**Fig 3:**
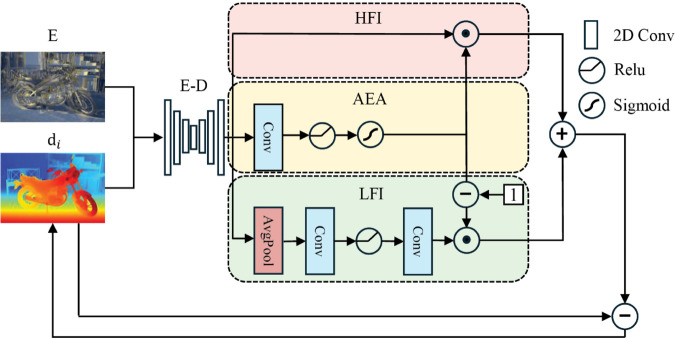
High and low-frequency separated disparity reconstruction module.

As shown in Fig 3, we input the iteratively updated disparity map into a lightweight Encoder-Decoder (E-D) network to obtain multi-scale and multi-channel disparity maps. Our HLFS module is primarily divided into three branches. The upper branch is the High Frequency Information (HFI) branch, where we apply no convolution operations to preserve the high-frequency local detail information in the original high-resolution disparity map. The middle branch is the Adaptive Error Adjustment (AEA) branch, which generates an adaptive weight map through a series of convolution operations followed by an activation function, used to adjust the proportions of high- and low-frequency errors in the error map. The lower branch is the Low Frequency Information (LFI) branch, where adaptive average pooling serves as a low-frequency filter to extract low-frequency information from the image while attenuating high-frequency information to some extent. By employing HLFS for disparity refinement, we can obtain a full-resolution refined disparity map. The specific implementation of HLFS is represented as:

$$o=ED\left(Concat\left(d_{n}^{\prime },E\right)\right)$$ (12)

HFI(o)=o⊙AEA(o) (13)

$$d_{n}=d_{n}^{\prime }-2DConv\left(LFI\left(o\right)⊙\left(1-AEA\left(o\right)\right)+HFI\left(o\right)\right)$$ (14)

where ⊙ is the Hadamard product, and $d_{n}^{\prime }$ is the disparity map before refinement.

### 3.4 Loss function

The overall loss of MGFD-Stereo consists of two parts. The first part is the initial disparity loss, which uses the Smooth L1 loss function to measure the error between the initial disparity and the ground truth disparity. Its expression is as follows:

$$L_{init}={\text{Smooth}}_{L1}\left(d_{0}-d_{gt}\right)$$ (15)

where *d*_0_ is the initial disparity value, *d*_*gt*_ is the ground truth disparity value, and the Smooth L1 loss is defined as follows:

$${\text{Smooth}}_{L1}(x)=\left\{ \begin{array}{cc} 0.5x^{2}, & \text{if }|x|<1\\ |x|-0.5, & \text{otherwise} \end{array}\right.$$ (16)

The second part of the loss is the error between the multistage predicted disparity and the true disparity, which is expressed as follows:

$$L_{iter}=\sum_{i=1}^{N}\gamma^{N-i}{\left\|d_{i}-d_{gt}\right\|}_{1}$$ (17)

Where *N* is the number of disparity iterations and γ=0.9. Therefore, the total loss function is expressed as follows:

$$L_{S}=L_{init}+L_{iter}$$ (18)

## 4 Experiment

### 4.1 Datasets and evaluation metrics

To test the performance of the proposed algorithm, we evaluate it on four publicly available datasets, Scene Flow [9], KITTI2012 [21], KITTI2015 [22], ETH3D [50] and Middlebury [51].

Scene Flow [9] is a synthetic stereo dataset containing 35,454 pairs of training samples and 4,370 pairs of testing samples, each with detailed dense disparity maps. SceneFlow includes two series of datasets: Cleanpass and Finalpass. This experiment uses the Finalpass dataset because it is closer to real-world images, making it more suitable for training and evaluating model performance. The experiment employs two metrics to assess the algorithm’s performance: the End-Point Error (EPE) and the three-pixel error percentage (D1).

KITTI 2012 [21] and KITTI 2015 [22] are datasets of real driving scenarios. KITTI 2012 includes 194 training image pairs and 195 testing image pairs, while KITTI 2015 includes 200 training image pairs and 200 testing image pairs. For the KITTI 2012 dataset, this experiment employs the percentage of erroneous pixels and the average end point error (EPE) in both all pixel regions (all) and non-occluded regions (noc) as evaluation metrics. For the KITTI 2015 dataset, the experiment adopts the three-pixel error percentages for background pixels (D1-bg), foreground pixels (D1-fg), and all pixels (D1-all), evaluated in both all pixel regions and non-occluded pixel regions, as evaluation metrics.

ETH3D [50] is a grayscale image dataset comprising 27 training image pairs and 20 testing image pairs. Middlebury 2014 [51] is an indoor scene dataset which consists of 15 sets of training image pairs and 15 sets of test image pairs, all of which are available in three different resolution formats. The ETH3D dataset uses the 1-pixel error as the experimental evaluation metric, while the Middlebury 2014 dataset uses the 2-pixel error as the experimental evaluation metric.

### 4.2 Implementation details

MGFD-Stereo is implemented using PyTorch framework and experimented on NVIDIA RTX 4090 GPU. In the experiments, this paper uses AdamW optimizer and crops the gradient to the range of [–1,1] for all the training datasets for brightness, colour saturation, scaling and flipping data. When training the MGFD-Stereo model on the Scene Flow dataset, the batch size is set to 8, the image is randomly cropped to 320 in height and 736 in width, the learning rate is 0.0002, and the training step size is 200k, and the model performance is monitored using the Scene Flow validation set. After that, the pre-trained model was fine-tuned on the KITTI2012 and KITTI2015 datasets by setting the batch size to 8, image random cropping height to 320, width to 736, learning rate to 0.0002, and training step size to 30k.

### 4.3 Ablation study

In order to better validate our proposed model structure, we conducted a large number of ablation experiments on the Scene Flow dataset, with all hyper-parameters set the same as the pre-training, and all the iteration rounds of the ablation experiments are 32, and the experimental results are shown in Table 1.

**Table 1:** Ablation experiments on the Scene Flow dataset.

Model			EPE(px)↓	D1(%)↓
MGI	FCAG	FLFS		
			0.53	2.78
✓			0.50	2.67
✓	✓		0.49	2.56
✓	✓	✓	**0.45**	**2.49**

The experimental results show that, when no additional modules were incorporated, the baseline model achieved an EPE of 0.53 px and a D1 error rate of 2.78%. Firstly, the introduction of the MGI module reduces the EPE to 0.50px and the D1 error rate to 2.67%, indicating that the MGI enables the cost volume to capture the geometric information better and improves the accuracy of the disparity estimation. The FCAG module is then added to the baseline model, and compared to the baseline model, the EPE decreases by 7.55% and the D1 error rate decreases by 7.91%, which fully demonstrates the effectiveness of our proposed module. Finally, we added the MGI, FCAG, and FLFS modules to the baseline model at the same time, which led to the optimal performance of the baseline model, with the EPE and D1 error rates of 0.45px and 2.49%, respectively, demonstrating that our FLFS module is able to correct the disparity maps further, resulting in a more refined full-resolution disparity map. This series of experimental results not only validates the effectiveness of each proposed module, but also shows that there is a synergistic effect between them, which together form an end-to-end accurate stereo matching framework.

To validate the effectiveness of the high- and low-frequency branches in the HFLS module, we conducted ablation studies on the Scene Flow dataset. All hyperparameter settings for the ablation studies were identical to those used during pretraining. As shown in Table 2, we separately evaluated the performance of configurations without the HLFS module, with only the low-frequency branch (HLFS-LFI), with only the high-frequency branch (HLFS-HFI), and with the complete HLFS module. The experimental results show that when only LFI is retained, EPE does not decrease while D1 decreases slightly, indicating that low-frequency information plays a fundamental role in modeling the overall structure of the disparity map. Because LFI attenuates part of the high-frequency errors,the resulting performance improvement of the model is limited. When retaining only the HFI, the EPE is reduced by 0.02 px, indicating that high-frequency information plays an important role in refining details and correcting edges. High-frequency information typically captures local image details and is beneficial for both deblurring and denoising. Conversely, low-frequency information generally represents the global structure of the image, aiding the description of background and contours. Therefore, separating high- and low-frequency errors and applying targeted corrections can enhance the accuracy of disparity maps.

**Table 2:** Ablation experiments of HLFS on the Scene Flow dataset.

Model	EPE(px)	D1(%)
HLFS-No	0.49	2.56
HLFS-LFI	0.49	2.54
HLFS-HFI	0.47	2.51
HLFS	**0.45**	**2.49**

During the fine-tuning process, we designed a series of ablation experiments to verify the effects of different settings and to obtain optimal performance. Two fine-tuning strategies were adopted: (1) Mixed fine-tuning, where the pre-trained model was fine-tuned using a combined training set from KITTI 2012 and KITTI 2015, with training steps of 30k, 40k, and 50k; (2) Independent fine-tuning, where the pre-trained model was fine-tuned on a single dataset, either the KITTI 2012 or KITTI 2015 training set, also with training steps of 30k, 40k, and 50k. The quantitative evaluation results are presented in Tables 3 and 4. Overall, increasing the number of fine-tuning steps does not necessarily yield better performance, as excessively long training tends to degrade the generalization capability of the method. For example, in the KITTI 2015 test results, both mixed and independent fine-tuning achieved the best performance at 30k training steps, while performance declined at 40k and 50k steps. Furthermore, mixed fine-tuning generally outperformed independent fine-tuning. This can be attributed to the limited size of individual datasets, which increases the risk of model overfitting. KITTI 2012 mainly contains static scenes, while KITTI 2015 includes a large number of dynamic objects. Thus, the two datasets are complementary in terms of data distribution and scene characteristics. By employing mixed fine-tuning, the diversity of training samples is increased, thereby improving the robustness of the model. A comprehensive analysis of the experimental results indicates that employing mixed fine-tuning with 30k training steps yields excellent performance on both test sets.

**Table 3:** Quantitative evaluation of Independent fine-tuning (2015) and Mixed fine-tuning (2012/15) on KITTI 2015.

Setting	KITTI-2015					
	All pixels (%)			Noc pixels (%)		
	D1-bg	D1-fg	D1-all	D1-bg	D1-fg	D1-all
2015-30k	1.55	**2.46**	1.70	1.42	**2.42**	1.59
2015-40k	1.56	2.62	1.74	1.44	2.62	1.64
2015-50k	1.55	2.70	1.74	1.42	2.66	1.62
2012/15-30k	**1.39**	2.54	**1.58**	1.29	2.52	**1.49**
2012/15-40k	1.41	2.59	1.61	**1.28**	2.54	1.49
2012/15-50k	1.42	2.57	1.61	1.29	2.53	1.49

**Table 4:** Quantitative evaluation of Independent fine-tuning (2012) and Mixed fine-tuning (2012/15) on KITTI 2012.

Setting	KITTI-2012							
	All				Reflective			
	Out-Noc(%)	Out-All(%)	Avg-Noc(px)	Avg-All(px)	Out-Noc(%)	Out-All(%)	Avg-Noc(px)	Avg-All(px)
2012-30k	1.17	1.50	0.4	0.5	4.56	5.28	1.1	1.1
2012-40k	1.21	1.55	0.4	0.5	4.43	5.11	1.0	1.0
2012-50k	1.22	1.55	0.4	0.5	4.66	5.43	1.0	1.1
2012/15-30k	**1.15**	**1.48**	**0.4**	**0.5**	**3.75**	**4.53**	**0.9**	**1.0**
2012/15-40k	1.16	1.50	0.4	0.5	4.38	5.29	0.9	1.0
2012/15-50k	1.17	1.51	0.4	0.5	4.23	5.14	0.9	1.0

### 4.4 Comparisons with state-of-the-art

To further validate the performance of the method, we compare it with other state-of-the-art methods on the datasets Scene Flow, KITTI2012, KITTI2015, ETH3D and Middlebury.

Table 5 shows the performance comparison between our proposed method and existing advanced stereo matching methods on the Scene Flow dataset. As shown in the table, our method achieves an end-point error of 0.45px, representing error reductions of 58.7% and 40.8% compared to classic stereo matching methods PSMNet [6] and GwcNet [15], respectively. Compared to recent state-of-the-art methods such as RAFT-Stereo [16], ACVNet [35], IGEV-Stereo [28], and DLNR [18], our method still achieves certain performance improvements. In particular, it reduces the error by approximately 4.3% compared to the current state-of-the-art IGEV-Stereo. These experimental results convincingly demonstrate the effectiveness and robustness of our method on synthetic scene datasets.

**Table 5:** Comparison with state-of-the-art methods on the Scene Flow dataset.

Method	PSMNet	GwcNet	RAFT-Stereo	ACVNet	IGEV-Stereo	DLNR	Ours
EPE(px)	1.09	0.76	0.54	0.48	0.47	0.48	**0.45**

To validate the performance of our method in real driving scenarios, we conducted benchmark tests on the KITTI2015 and KITTI2012 datasets, with results shown in Tables 6 and 7. In the KITTI2015 stereo matching benchmark, our method was compared with state-of-the-art stereo matching approaches including PSMNet [6], GwcNet [15], CFNet [23], RAFT-Stereo [16], CREStereo [42], IGEV-Stereo [28], DFMNet [30], LoS [31] and DLNR [18]. Our method achieves D1-bg and D1-fg error rates of 1.39% and 2.54% respectively across all pixel regions, demonstrating outstanding performance. This indicates that our approach delivers highly accurate depth estimation in both static and dynamic areas of the scene, highlighting its robustness and generalization capability. Our method attains low error rates when processing both all-pixel regions and non-occluded pixel regions, with minimal difference between these error rates, indicating that the method exhibits robustness when handling occluded regions.

**Table 6:** Quantitative evaluation on KITTI 2015.

Method	KITTI-2015						
	All pixels (%)			Noc pixels (%)			Time/s
	D1-bg	D1-fg	D1-all	D1-bg	D1-fg	D1-all	
PSMNet	1.86	4.62	2.32	1.71	4.31	2.14	0.41
GwcNet	1.74	3.93	2.11	1.61	3.49	1.92	0.32
CFNet	1.54	3.56	1.88	1.43	3.25	1.73	**0.18**
RAFT-Stereo	1.58	3.05	1.82	1.45	2.94	1.69	0.38
CREStereo	1.45	2.86	1.69	1.33	2.60	1.54	0.41
IGEV-Stereo	**1.38**	2.67	1.59	**1.27**	2.62	1.49	0.18
DFMNet	1.39	3.12	1.75	-	-	-	0.20
LoS	1.42	2.81	1.65	1.29	2.66	1.52	0.19
DLNR	1.60	2.59	1.76	1.45	**2.39**	1.61	-
Ours	1.39	**2.54**	**1.58**	1.29	2.52	**1.49**	0.35

**Table 7:** Quantitative evaluation on KITTI 2012.

Method	KITTI-2012							
	All				Reflective			
	Out-Noc(%)	Out-All(%)	Avg-Noc(px)	Avg-All(px)	Out-Noc(%)	Out-All(%)	Avg-Noc(px)	Avg-All(px)
PSMNet	1.49	1.89	0.5	0.6	8.36	10.18	1.4	1.6
GwcNet	1.32	1.70	0.5	0.5	7.80	9.28	1.3	1.4
RAFT-Stereo	1.30	1.66	0.4	0.5	5.40	6.48	1.3	1.3
CREStereo	1.14	1.46	0.4	0.5	6.27	7.27	1.4	1.4
IGEV-Stereo	1.12	**1.44**	0.4	**0.4**	4.35	5.00	1.0	1.1
DVANet	**1.09**	1.52	0.4	0.5	4.39	4.62	-	-
DFMNet	1.14	1.48	0.4	0.5	-	-	-	-
IVF-AStereo	1.52	1.87	0.5	0.6	-	-	-	-
Ours	1.15	1.48	**0.4**	0.5	**3.75**	**4.53**	**0.9**	**1.0**

In addition, we conducted quantitative comparisons with state-of-the-art methods including PSMNet [6], GwcNet [15], RAFT-Stereo [16], CREStereo [42], IGEV-Stereo [28], DFMNet [30], IVF-AStereo [32] and DVANet [52] on both the all regions and reflective regions of the KITTI 2012 test set. Experimental results show that while our method did not achieve optimal performance across all metrics in the all regions, it remains highly competitive compared to the state-of-the-art methods. However, our method achieves the best performance across all metrics in reflective regions, indicating superior robustness and accuracy when handling complex scenes and reflective regions. Our method focuses on edge details and complex scenes, while disparity optimization in regular regions is relatively limited. Therefore, the metrics in some regions are not optimal.

To further evaluate the generalization capability of our method, we compared its performance on the ETH3D and Middlebury datasets, with results shown in Table 8. Although our method did not achieve optimal results on the Middlebury and ETH3D datasets, it demonstrates excellent performance across different resolutions and scenes, showing competitive capability against advanced methods.

**Table 8:** Quantitative evaluation on Middlebury and ETH3D.

Method	Middlebury		ETH3D
	Half	Quarter	
PSMNet	15.8	9.8	10.2
GANet	13.5	8.5	6.5
CFNet	15.3	9.8	5.8
RAFT-Stereo	8.7	7.3	**3.2**
DLNR	9.5	7.6	23.1
IGEV-Stereo	**7.1**	**6.2**	3.6
Ours	7.3	6.4	3.5

### 4.5 Visualization results and analysis

**Fig 4:**
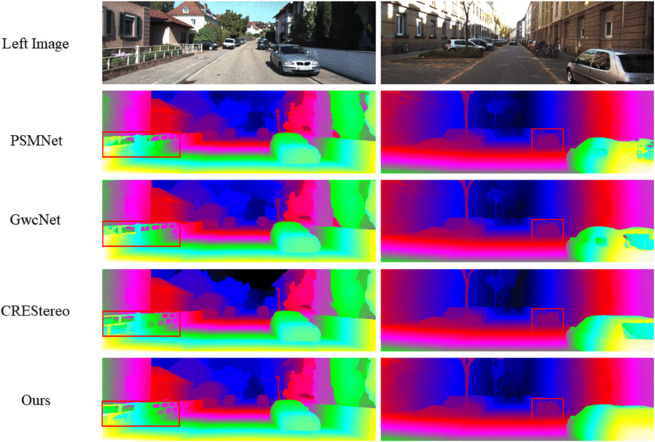
Visualization Results on the KITTI2012 Dataset.

**Fig 5:**
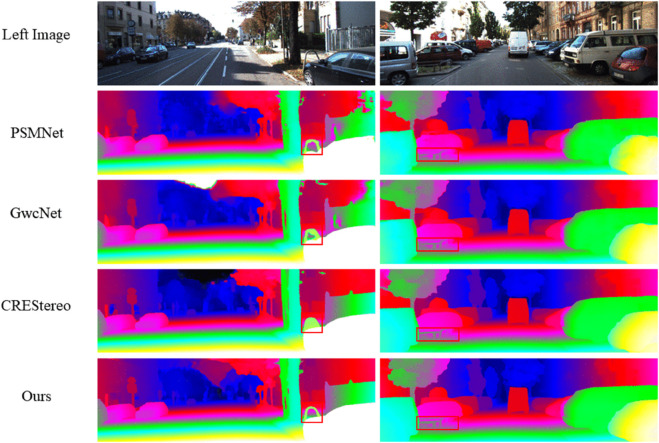
Visualization Results on the KITTI2015 Dataset.

In order to visually verify the effectiveness of the proposed method and deeply understand its performance in different scenarios, we conducted detailed visualisation results and analysis. In the datasets KITTI2012 and KITTI2015, we compare our method with state-of-the-art methods PSMNet [6], GwcNet [15], and CREStereo [42], as shown in Figs 4 and 5. The visualisation results show that our method is able to accurately capture the detailed regions in the scene, such as fences, parking stakes, and vehicle edges, which suggests that our method’s performance in matching edge details is excellent and that our method can accurately match the background and foreground regions of the scene to avoid confusion.

**Fig 6:**
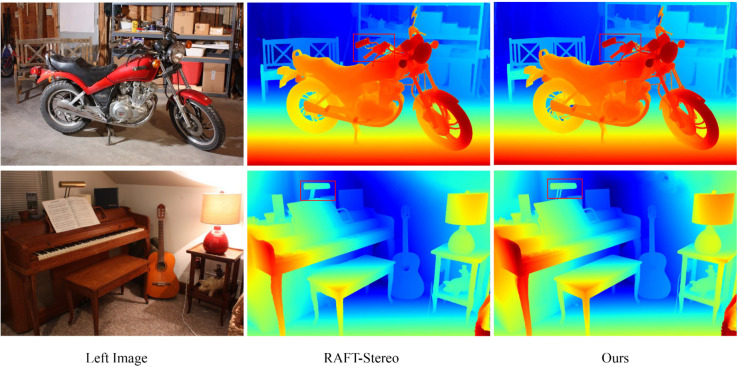
Visualization Results on the Middlebury Dataset.

In the dataset Middlebury, we compared with the state-of-the-art method RAFT-Stereo [16], as shown in Fig 6. Compared with RAFT-Stereo [16], our method is able to better preserve the detail information, effectively solving the problem of matching blurring at the edges and details, and the disparity maps estimated by our method are smoother and more coherent, with a significant improvement in detail prediction.

## 5 Conclusion

We propose a learning-based stereo matching network that integrates multi-scale geometric features with a frequency-domain decomposition strategy. The cost volume is processed by the multi-scale geometric extraction module, which effectively solves the problem of the lack of geometric information in the cost volume, which leads to the confusion of foreground and background disparity estimation, and the blurring at the edges and details. The adaptive guidance mechanism based on channel attention significantly improves the quality of cost aggregation while maintaining computational efficiency. Our proposed frequency-domain decomposition disparity reconstruction method is able to obtain finer disparity maps by handling high and low frequency errors separately. Experiments on the Scene Flow, KITTI2012, and KITTI2015 datasets show that our method achieves strong performance and performs well on the ETH3D and Middlebury datasets, indicating good cross-dataset generalisation.

## References

[1] Xu G, Wang X, Zhang Z, Cheng J, Liao C, Yang X. IGEV++: iterative multi-range geometry encoding volumes for stereo matching. IEEE Trans Pattern Anal Mach Intell. 2025;47(8):7108–22. doi: 10.1109/TPAMI.2025.3569218 40354217

[2] Li J, Gao W, Wu Y, Liu Y, Shen Y. High-quality indoor scene 3D reconstruction with RGB-D cameras: a brief review. Comp Visual Med. 2022;8(3):369–93. doi: 10.1007/s41095-021-0250-8

[3] Xia W, Chen ECS, Pautler S, Peters TM. A robust edge-preserving stereo matching method for laparoscopic images. IEEE Trans Med Imaging. 2022;41(7):1651–64. doi: 10.1109/TMI.2022.3147414 35085075

[4] Lv Z, Lloret J, Song H. Real-time image processing for augmented reality on mobile devices. J Real-Time Image Proc. 2021;18(2):245–8. doi: 10.1007/s11554-021-01097-9

[5] Yang G, Song X, Huang C. DrivingStereo: a large-scale dataset for stereo matching in autonomous driving scenarios. In: Proceedings of the IEEE/CVF Conference on Computer Vision and Pattern Recognition (CVPR). 2019. p. 899–908.

[6] Chang JR, Chen YS. Pyramid stereo matching network. In: Proceedings of the IEEE Conference on Computer Vision and Pattern Recognition (CVPR). 2018. p. 5410–8.

[7] Scharstein D, Szeliski R, Zabih R. A taxonomy and evaluation of dense two-frame stereo correspondence algorithms. In: Proceedings IEEE Workshop on Stereo and Multi-Baseline Vision (SMBV 2001). p. 131–40. 10.1109/smbv.2001.988771

[8] Zbontar J, LeCun Y. Computing the stereo matching cost with a convolutional neural network. In: Proceedings of the IEEE Conference on Computer Vision and Pattern Recognition (CVPR); 2015. p. 1592–9.

[9] Mayer N, Ilg E, Hausser P. A large dataset to train convolutional networks for disparity, optical flow, and scene flow estimation. In: Proceedings of the IEEE Conference on Computer Vision and Pattern Recognition (CVPR). 2016. p. 4040–8.

[10] Xu H, Zhang J. AANet: adaptive aggregation network for efficient stereo matching. In: Proceedings of the IEEE/CVF Conference on Computer Vision and Pattern Recognition (CVPR). 2020. p. 1959–68.

[11] Tankovich V, Hane C, Zhang Y. HITNet: hierarchical iterative tile refinement network for real-time stereo matching. In: Proceedings of the IEEE/CVF Conference on Computer Vision and Pattern Recognition (CVPR). 2021. p. 14362–72.

[12] Badki A, Troccoli A, Kim K. Bi3D: stereo depth estimation via binary classifications. In: Proceedings of the IEEE/CVF Conference on Computer Vision and Pattern Recognition (CVPR). 2020. p. 1600–8.

[13] Tosi F, Liao Y, Schmitt C. SMD-Nets: stereo mixture density networks. In: Proceedings of the IEEE/CVF Conference on Computer Vision and Pattern Recognition (CVPR). 2021. p. 8942–52.

[14] Kendall A, Martirosyan H, Dasgupta S, Henry P, Kennedy R, Bachrach A, et al. End-to-end learning of geometry and context for deep stereo regression. In: 2017 IEEE International Conference on Computer Vision (ICCV). 2017. p. 66–75. 10.1109/iccv.2017.17

[15] Guo X, Yang K, Yang W. Group-wise correlation stereo network. In: Proceedings of the IEEE/CVF Conference on Computer Vision and Pattern Recognition (CVPR). 2019. p. 3273–82.

[16] Lipson L, Teed Z, Deng J. RAFT-stereo: multilevel recurrent field transforms for stereo matching. In: 2021 International Conference on 3D Vision (3DV). 2021. p. 218–27. 10.1109/3dv53792.2021.00032

[17] Cho K, Van Merriënboer B, Gulcehre C. Learning phrase representations using RNN encoder-decoder for statistical machine translation. 2014.

[18] Zhao H, Zhou H, Zhang Y, et al. High-frequency stereo matching network. In: Proceedings of the IEEE/CVF Conference on Computer Vision and Pattern Recognition (CVPR); 2023. p. 1327–36.

[19] Hochreiter S, Schmidhuber J. Long short-term memory. Neural Comput. 1997;9(8):1735–80. doi: 10.1162/neco.1997.9.8.1735 9377276

[20] Wang X, Xu G, Jia H. Selective-stereo: adaptive frequency information selection for stereo matching. In: Proceedings of the IEEE/CVF Conference on Computer Vision and Pattern Recognition (CVPR). 2024. p. 19701–10.

[21] Geiger A, Lenz P, Urtasun R. Are we ready for autonomous driving? The KITTI vision benchmark suite. In: 2012 IEEE Conference on Computer Vision and Pattern Recognition. 2012. p. 3354–61. 10.1109/cvpr.2012.6248074

[22] Menze M, Geiger A. Object scene flow for autonomous vehicles. In: Proceedings of the IEEE Conference on Computer Vision and Pattern Recognition (CVPR); 2015. p. 3061–70.

[23] Zhang K, Li D, Luo W, Ren W, Liu W. Enhanced spatio-temporal interaction learning for video deraining: faster and better. IEEE Trans Pattern Anal Mach Intell. 2023;45(1):1287–93. doi: 10.1109/TPAMI.2022.3148707 35130145

[24] Zhang K, Li R, Yu Y, Luo W, Li C. Deep dense multi-scale network for snow removal using semantic and depth priors. IEEE Trans Image Process. 2021;30:7419–31. doi: 10.1109/TIP.2021.3104166 34403338

[25] Wang T, Zhang K, Shao Z, Luo W, Stenger B, Lu T, et al. GridFormer: residual dense transformer with grid structure for image restoration in adverse weather conditions. Int J Comput Vis. 2024;132(10):4541–63. doi: 10.1007/s11263-024-02056-0

[26] Jin Z, Qiu Y, Zhang K, Li H, Luo W. MB-TaylorFormer V2: improved multi-branch linear transformer expanded by taylor formula for image restoration. IEEE Trans Pattern Anal Mach Intell. 2025;47(7):5990–6005. doi: 10.1109/TPAMI.2025.3559891 40208767

[27] Shen Z, Dai Y, Rao Z. CFNet: cascade and fused cost volume for robust stereo matching. In: Proceedings of the IEEE/CVF Conference on Computer Vision and Pattern Recognition (CVPR); 2021. p. 13906–15.

[28] Xu G, Wang X, Ding X, et al. Iterative geometry encoding volume for stereo matching. In: Proceedings of the IEEE/CVF Conference on Computer Vision and Pattern Recognition (CVPR); 2023. p. 21919–28.

[29] Chen Z, Long W, Yao H. Mocha-stereo: motif channel attention network for stereo matching. In: Proceedings of the IEEE/CVF Conference on Computer Vision and Pattern Recognition (CVPR). 2024. p. 27768–77.

[30] Lin S, Zhuo X, Qi B. Accuracy and efficiency stereo matching network with adaptive feature modulation. PLoS One. 2024;19(4):e0301093. doi: 10.1371/journal.pone.0301093 38662662 PMC11045109

[31] Li K, Wang L, Zhang Y, et al. LoS: local structure-guided stereo matching. In: Proceedings of the IEEE/CVF Conference on Computer Vision and Pattern Recognition (CVPR); 2024. p. 19746–56.

[32] Gao Y, Shen L. Iterative volume fusion for asymmetric stereo matching. In: 2025 IEEE International Conference on Robotics and Automation (ICRA). 2025. p. 10907–14. 10.1109/icra55743.2025.11127981

[33] Li Z, Liu X, Drenkow N. Revisiting stereo depth estimation from a sequence-to-sequence perspective with transformers. In: Proceedings of the IEEE/CVF International Conference on Computer Vision (ICCV). 2021. p. 6177–86.

[34] Vaswani A, Shazeer N, Parmar N, et al. Attention is all you need. In: Advances in Neural Information Processing Systems. 2017. https://proceedings.neurips.cc/paper/2017/hash/3f5ee243547dee91fbd053c1c4a845aa-Abstract.html

[35] Xu G, Cheng J, Guo P, et al. Attention concatenation volume for accurate and efficient stereo matching. In: Proceedings of the IEEE/CVF Conference on Computer Vision and Pattern Recognition (CVPR); 2022. p. 12981–90.

[36] Cheng X, Wang P, Yang R. Depth estimation via affinity learned with convolutional spatial propagation network. In: Proceedings of the European Conference on Computer Vision (ECCV); 2018. p. 103–19.

[37] Zhang F, Prisacariu V, Yang R. GA-Net: guided aggregation net for end-to-end stereo matching. In: Proceedings of the IEEE/CVF Conference on Computer Vision and Pattern Recognition (CVPR). 2019. p. 185–94.

[38] Khamis S, Fanello S, Rhemann C, Kowdle A, Valentin J, Izadi S. StereoNet: guided hierarchical refinement for real-time edge-aware depth prediction. Lecture Notes in Computer Science. Springer International Publishing. 2018. p. 596–613. 10.1007/978-3-030-01267-0_35

[39] Dai H, Zhang X, Zhao Y, Sun H, Zheng N. Adaptive disparity candidates prediction network for efficient real-time stereo matching. IEEE Trans Circuits Syst Video Technol. 2022;32(5):3099–110. doi: 10.1109/tcsvt.2021.3102109

[40] Li X, Zhang C, Su W, Tao W. IINet: implicit intra-inter information fusion for real-time stereo matching. AAAI. 2024;38(4):3225–33. doi: 10.1609/aaai.v38i4.28107

[41] Teed Z, Deng J. RAFT: recurrent all-pairs field transforms for optical flow. Lecture Notes in Computer Science. Springer; 2020. p. 402–19. 10.1007/978-3-030-58536-5_24

[42] Li J, Wang P, Xiong P. Practical stereo matching via cascaded recurrent network with adaptive correlation. In: Proceedings of the IEEE/CVF Conference on Computer Vision and Pattern Recognition (CVPR). 2022. p. 16242–51.

[43] Chabra R, Straub J, Sweeney C. StereoDRNet: dilated residual StereoNet. In: Proceedings of the IEEE/CVF Conference on Computer Vision and Pattern Recognition (CVPR). 2019. p. 11786–95.

[44] Zhao H, Zhou H, Zhang Y, et al. EAI-stereo: error aware iterative network for stereo matching. In: Proceedings of the Asian Conference on Computer Vision (ACCV); 2022. p. 315–32. 10.1007/978-3-031-26319-4_1

[45] Chen G, Dai K, Yang K, et al. Bracketing image restoration and enhancement with high-low frequency decomposition. In: Proceedings of the IEEE/CVF Conference on Computer Vision and Pattern Recognition (CVPR); 2024. p. 6097–107.

[46] Wang J, Liang Y, Yang X, Zhao M, Xia J. HiLoF-GAN: high and low frequency information separation GAN for underwater image enhancement. Earth Sci Inform. 2025;18(4):1–19. doi: 10.1007/s12145-025-02020-7

[47] Pi R, Xu J, Peng Y. FE-VAD: high-low frequency enhanced weakly supervised video anomaly detection. In: 2024 IEEE International Conference on Multimedia and Expo (ICME). 2024. p. 1–6.

[48] Sandler M, Howard A, Zhu M. MobileNetV2: inverted residuals and linear bottlenecks. In: Proceedings of the IEEE Conference on Computer Vision and Pattern Recognition (CVPR). 2018. p. 4510–20.

[49] Deng J, Dong W, Socher R, Li L-J, Kai Li, Li Fei-Fei. ImageNet: a large-scale hierarchical image database. In: 2009 IEEE Conference on Computer Vision and Pattern Recognition. 2009. p. 248–55. 10.1109/cvpr.2009.5206848

[50] Schöps T, Schönberger JL, Galliani S. A multi-view stereo benchmark with high-resolution images and multi-camera videos. In: Proceedings of the IEEE Conference on Computer Vision and Pattern Recognition (CVPR). 2017. p. 3260–9.

[51] Scharstein D, Hirschmüller H, Kitajima Y, Krathwohl G, Nešić N, Wang X, et al. High-resolution stereo datasets with subpixel-accurate ground truth. Lecture Notes in Computer Science. Springer; 2014. p. 31–42. 10.1007/978-3-319-11752-2_3

[52] Zhao T, Ding M, Zhan W. Type. arXiv preprint 2024. https://arxiv.org/abs/2402.08931

